# Recent Advances in Ophthalmic Science

**Published:** 1866-08

**Authors:** 


Recent Advances in Ophthalmic Science. The Boylston Prize Essay for 1865. By Henry W. Williams, M.D., Ophthalmic Surgeon to the City Hospital, Boston; University Lecturer on Ophthalmic Surgery, in Harvard University; etc., etc. Ticknor & Fields. Boston. 1866.

This is an elegantly published volume of 166 pages. The title sufficiently indicates the interesting and important nature of its contents; while the reputation of its author and the award of the prize committee are a sufficient guarantee that the topics are well presented.

